# Collection of Post-treatment PRO Data in Oncology Clinical Trials

**DOI:** 10.1007/s43441-020-00195-3

**Published:** 2020-07-08

**Authors:** J. Jason Lundy, Cheryl D. Coon, An-Chen Fu, Vivek Pawar

**Affiliations:** 1 Outcometrix, St. Petersburg, FL USA; 2 EMD Serono Research & Development Institute, Inc., Billerica, MA USA; 3 Outcometrix, 433 Central Avenue, Suite 300, St. Petersburg, FL 33701 USA

**Keywords:** Post-treatment data, Long-term follow-up, Patient-reported outcome, Oncology clinical trials, Cancer

## Abstract

As patient-reported outcome (PRO) measures are being included more frequently in oncology clinical trials, regulatory and health technology assessment agencies have begun to request long-term, post-treatment PRO data to supplement traditional survival/progression endpoints. These data may be collected as part of cohort extension or registry studies to describe long-term outcomes of study participants after concluding their cancer treatment. While post-treatment PRO data may be expected to satisfy regulatory and payer expectations, significant practical barriers exist for the efficient incorporation of these data into oncology clinical trials, such as subject attrition, protocol deviations, and treatment crossover. The incorporation of post-treatment PRO assessments is a resource-intensive task requiring clear objectives for how the data will be analyzed and interpreted by both sponsors and regulators. Incorporating PRO data collection via electronic modalities (e.g., smartphone, web) may be a less expensive and more feasible option for incorporating long-term follow-up, reducing the frequency of manual study staff follow-up and expensive clinic visits. It is essential to include well-defined estimands for the statistical analysis, as well as to document limitations associated with the long-term follow-up data-collection approach. Analytical techniques will likely rely on descriptive and model-based statistics, and conclusions about treatment differences will likely be limited to preliminary findings of effectiveness (instead of efficacy). Finally, communications with health authorities and regulatory agencies regarding the LTFU study design and analysis should occur as early as possible to ensure that the PRO data to be collected offer an opportunity to properly evaluate the research question(s) of interest.

## Introduction

As patient-reported outcome (PRO) measures are being included more frequently in oncology clinical trials, regulatory and health technology assessment (HTA) agencies have begun to request long-term, post-treatment PRO data to supplement the traditional survival/progression endpoints. For instance, Germany’s Federal Joint Committee (German: *Gemeinsamer Bundesausschuss* [G-BA]) requests efficacy data for new cancer drugs, including assessments of mortality, morbidity, and health-related quality of life (HRQOL), with specific recommendations to collect post-progression (an example of a long-term post-treatment scenario) HRQOL data [1]. PRO data were key in G-BA’s decision of assigning an additional benefit rating to crizotinib, and payers have noted the importance of post-progression PRO data in differentiating new oncology drugs [2]. The importance of post-treatment PRO assessments is further highlighted by the American Society of Clinical Oncology recommendations for cancer survivors experiencing chronic pain, which calls for screening for pain at each visit and for evaluating and monitoring for “recurrent disease, second malignancy, or late-onset treatment effects in any patient who reports new-onset pain” [3].

In addition, the European Medicines Agency’s (EMA’s) reflection paper for collecting PROs in oncology suggests that sponsors collect post-treatment PROs in an attempt to balance follow-up data between comparator groups (i.e., theoretical equipoise). In addition, the United States Food and Drug Administration (FDA) has promoted the collection of PRO data in oncology studies [4], and both the FDA and EMA require long-term follow-up (LTFU) data for gene therapy studies, including some oncology therapies (e.g., CAR T-cell therapy). Additionally, Cuzick [5], a leading cancer researcher and statistician, has suggested collecting follow-up data for all participants in oncology studies until drop out or death. These LTFU data would ideally allow for comparisons of effectiveness rather than just efficacy, detection of late occurrence of efficacy and/or adverse events, and better estimation of recurrence. However, due to the recency of these recommendations and the focus on clinical events (e.g., survival, safety), it is not clear how common LTFU PRO data collection is and what approach sponsors are taking to incorporate it into oncology studies.

Further complicating this topic, the definitions of “long-term” and “post-treatment” time periods are unclear in the literature and variable across clinical trials. In a review of Original Reports in the *Journal of Clinical Oncology,* Bentensky [6] concluded that “the concept of follow-up is ill-defined in reports of clinical studies.” Defining the start of the “post-treatment period” is not straightforward and could result in different time periods being considered, not only between trials, but even between participants in the same trial. For example, the “post-treatment period” could begin at the cessation of the assigned treatment for an individual participant (e.g., due to progression or treatment crossover), the co-administration of another therapy while the participant is still taking the assigned treatment, or the end of the study treatment period as defined in the clinical trial protocol for the entire group of participants (e.g., week 13). Furthermore, how the “post-treatment” time period is determined also may impact comparative analyses, as variations in lengths of follow-up will lead to missing data affecting the comparability of certain endpoints and outcomes. As many analytical approaches depend on assumptions regarding the nature of missing data (e.g., missing at random assumptions, missing completely at random assumption), violations of such assumptions could impact the validity of the analyses.

Despite the various interpretations of the terms “long term” and “post treatment,” this type of PRO data is becoming more relevant in the eyes of stakeholders evaluating oncology drugs. While there are many potential benefits of collecting PRO data in oncology studies for participants no longer receiving study treatment, it comes with many practical and methodological challenges. As there is limited information in the literature to offer guidance for the collection and analysis of PRO data in this context, the objective of this commentary is to identify the challenges of collecting post-treatment PRO data in oncology clinical trials and offer potential methodological and analytical solutions to overcome these challenges.

## Discussion

### Barriers to Post-treatment Assessment in Oncology Studies

One of the central issues affecting feasibility of post-treatment follow-up is increased subject attrition over time. There are many reasons for subject attrition in clinical trials, but in oncology studies, this is mostly due to disease progression and death. Trials with patients whose disease has progressed may be more prone to attrition because the patients may not have the energy or interest to respond to a PRO measure; however, there is evidence that patients with advanced cancers are willing to complete PRO measures to facilitate to further research on their disease [7]. Disease progression may also result in the patient beginning a new course of treatment, and if the patient enters a new clinical trial, they may be unwilling to continue providing responses to PRO measures associated with both the prior study and the new clinical trial. To add to the complications, attrition due to disease progression and death will vary by the type and stage of the disease. For instance, in cancer types with positive prognoses and high survival rates, such as localized breast cancer, nearly all patients (99%) are expected to live at least 5 years [8], thereby making long-term, post-treatment PRO assessments more feasible and interpretable. However, in advanced cancer types, such as distant (e.g., stage IV) non-small cell lung cancer where the 5-year survival rate is 6% [8], only a small proportion of patients will likely survive long enough to be available for long-term data collection. Even with perfect compliance in this proportion of patients, the analysis of their PRO data would be restricted by the small sample size, perhaps rendering the PRO data inconclusive or uninterpretable.

A second barrier is deviations from the protocol, which are particularly problematic for analyses aimed at assessing efficacy (vs real-world effectiveness). PRO-related protocol deviations come in many forms but can be classified into 2 categories: site-related deviations and subject-related deviations. Site-related protocol deviations include the site staff failing to administer 1 or all of the PRO measures (e.g., due to forgetting, running out of time during the visit, or deciding the patient was too ill to complete the PRO); site staff administering the PRO measures in the wrong sequence or at the wrong visits; the site staff failing to charge the electronic PRO (ePRO) device; the ePRO device malfunctioning with no substitute available; and the site staff defaulting to paper-based PRO data collection when ePRO data collection was specified in the protocol, potentially raising questions of systematic bias due to the mixing of PRO administration modes. Subject-related protocol deviations are primarily due to the patient missing a scheduled assessment or clinic visit (with no follow-up procedure in place to capture these data) or the patient’s refusal to complete the PRO assessment at a given visit, which may be due to a poor or deteriorating health condition or the patient feeling that the PRO measures are too time-consuming, inconvenient, irrelevant, or unclear. When post-treatment assessments are completed outside of the study site, such as at home using online or telephone surveys, the patient may have difficulty remembering to complete the PRO or accessing the system within which to provide responses. Regardless of the source, these protocol deviations are particularly problematic when they occur during the baseline visit/assessment, which effectively excludes that patient’s data from being used in analyses of change from baseline and time until deterioration, the 2 most common PRO analyses in oncology clinical trials. Thus, protocol deviations leading to non-compliance compound problems associated with loss to follow-up, where both the attrition and non-compliance complicate the estimation of causal effects.

A third barrier is treatment crossover (or treatment switching), which complicates the analysis of LTFU data as the control participants may crossover to the experimental treatment arm (or vice versa), and/or participants may be administered concurrent treatments that are not under specific investigation in the trial. Similar to differential loss to follow-up based on disease stage, trial participants with poor outcomes may be more likely to receive co-interventions than those with good outcomes. This scenario could confound estimation of the intervention and may necessitate a sensitivity analysis to be able to attribute changes in symptoms and functioning to a specific treatment.

Finally, the collection of long-term, post-treatment data has produced results of limited clinical utility beyond those known from the clinical trial’s primary analysis, which is likely attributable to some of the barriers identified above. A systematic evaluation of oncology approvals by the EMA in 2009–2013 showed that at a minimum of 3.3 years after market, there was still no conclusive evidence that these drugs either extended or improved QOL for most cancer indications [9]. A systematic evaluation of accelerated approvals by the FDA in 2009–2013 found that of 38 required post-approval studies, only 7 (18%) included follow-up longer than 3 years [10]. These long-term assessments require additional resources on top of already constrained clinical trial budgets and timelines, and the added value of long-term, post-treatment PRO data is yet to be determined.

### Potential Solutions for Post-treatment Assessment in Oncology and Related Considerations

#### Frequency and Duration of PRO Administration

When designing a clinical trial protocol, the assessment frequency and duration of PRO instruments should be selected by considering the context of the population being evaluated, the measurement properties of the chosen instruments, and the way in which the resulting data will be used for assessing treatment efficacy and/or safety. PRO assessment frequency and duration decisions are even more complex in the post-treatment period than in the treatment period due to a number of factors, including the expectancy of patient survival after study treatment ends and treatment crossover (or treatment switching) as noted above. Based on findings in the literature, it appears that the times for LTFU PRO assessments in oncology clinical trials are highly variable. The intervals between administrations (1 week to 1 year) and the length of time for conducting post-baseline assessments (3 months to 5 years) vary greatly between trials [11–15]. In addition, no guidance is offered in the PRO literature for assessment frequency or duration of post-treatment PRO assessments. It seems reasonable to suggest continuing the same PRO assessment frequency as that used during the treatment period for the post-treatment assessment in situations where the patient does not immediately transition to an alternative treatment. It also seems reasonable that the duration of the post-treatment PRO data collection mirrors that of the treatment period. For example, if a clinical trial has a 12-week treatment period and the PRO instruments are administered at baseline and every 2 weeks through the end of the treatment visit at week 12 (i.e., PRO administration at baseline and weeks 2, 4, 6, 8, 10, and 12), the post-treatment assessment period would continue for 12 weeks and 2-week PRO assessment frequency would continue through week 24 (i.e., PRO administration at weeks 14, 16, 18, 20, 22, and 24). The advantage of this approach is that the same or similar statistical model used for the treatment data can be applied to the post-treatment data. This strategy would provide comparable data-collection opportunities during each time period. The duration of the post-treatment assessment time period can be extended beyond the above recommendation for cancers with high survival rates (e.g., early-stage breast cancer) and where efforts are made to limit subject attrition over time. It should be noted that the symptom and functioning experience of the patient becomes more distal to the treatment as the PRO assessment duration is extended. This has implications for the utility of these data in any decision to extend the PRO assessment duration as well as for how these data are analyzed and interpreted. The decision regarding the frequency and duration of the post-treatment PRO assessments should be made with the end in mind while considering how the data will be analyzed and interpreted for stakeholder communication. Finally, sponsors should consider data monitoring strategies to ensure data are being collected at the expected intervals, and if not, strategies are in place to follow-up with sites and/or patients to collect these data.

#### Site-Based Data Collection

All trials that aim to collect post-treatment data will need to incentivize sites and participants to report the data. Because there is little that can be done to overcome attrition of patients due to severe deterioration of health status and death, it is imperative that sponsors provide the proper incentives to reduce the loss to follow-up from those participants who are still able to provide PRO data. Site staff need to be informed of the importance of PRO data to future patients, their clinicians, and stakeholders, and they must be trained on proper data-collection protocols and on the implications of deviating from those protocols. To instill the importance of collecting these data, sponsors are encouraged to connect contract research organization (CRO) and site payments to the completeness of the per-protocol PRO data collected during various phases of the study. Furthermore, we should not expect patients to attend clinic visits or provide PRO data for no or minimal incentives. Similar completeness-based incentives could be employed for the participants, whereby the total monetary incentive earned increases the longer the participant provides PRO data.

#### Electronic Patient-Reported Outcome Data Collection

Concurrent with the increase in the use of PRO instruments in oncology clinical trials, electronic data-capturing platforms to collect patient-reported data have increasingly been employed. Electronically adapted PRO instruments (ePROs) have the advantages of less administrative burden, avoidance of secondary data entry errors, and more accurate and complete data [16–18]. These advances in electronic data capture should enhance the data-collection process for both investigators and participants and are particularly well suited for collecting LTFU data from the patient. The use of ePRO instruments, either as provisioned devices, bring your own device (BYOD), or a combination of both for LTFU studies may limit the need for expensive follow-up visits. While the details are outside the scope of this article, the use of smartphones, web applications, and interactive voice response (IVR) technologies offer an array of options for engaging participants in an efficient and timely manner. Further, the use of ePRO instruments allows the sponsor greater flexibility regarding the frequency and duration of post-treatment PRO data collection, thereby increasing those data’s utility in future analyses. It is important to provide adequate training to the study participant on the use of the electronic device, ensure the availability of web-based or telephone-based support (e.g., help-desk) for technological problems that may arise, and consider backup strategies for collecting data in the event of device failure.

#### Extension Studies

In addition to planning to reduce attrition, one of the strategies that can be employed to capture post-treatment PRO data is to pre-plan study extension (beyond the treatment period) for a select number of clinical trial participants. Limiting the number of participants and/or limiting the follow-up period beyond the end of treatment provides a way to limit cost, retain protocol integrity, and utilize strategies to keep response rates high. These extension studies are likely most useful in cancer types and stages with high survival rates that allow for longer-term follow-up due to low attrition rates. In cases where attrition due to disease progression or death is high, consider using ePRO modalities (either provisioned devices or the participant’s own device) and conduct assessments more frequently to optimize the collection of data from participants before they are lost to follow-up.

The venue for follow-up (e.g., passive follow-up via registries vs direct, active follow-up via cohort extension) should be considered, as the choice may impact the frequency and quality of PRO data collection. Further, the venue for data collection will be guided by whether the sponsor chooses to extend the follow-up period for the clinical trial cohort or conduct a separate study as in the case of a registry. In the case of registries, PRO data collection may be passively supplied via the treating physician, in which case the frequency of data collection may be highly variable. Data may also be limited to either the patients’ scheduled exams (e.g., semi-annual exams) or during visits for treatment of either their cancer or another health problem. In these latter cases, PRO data collected at these timepoints may be biased reports (increased symptoms or decreased function) of the patient’s general symptom and functional status. The choice of venue should balance what is pragmatic with how the data are intended to be used. For example, if longitudinal change from baseline modeling is desired, then active follow-up would provide greater control over the homogeneity of assessment time points across participants needed for statistical modeling. Conversely, if the LTFU PRO data are intended for descriptive reporting only, then registries with less control over assessment time points may suffice. Regardless of the venue, the importance of the PRO data must be conveyed to the site staff and study participants to ensure that they understand the value of participants’ responses to health authorities making treatment access decisions and cancer patients and clinicians making treatment decisions in the future.

#### Clarifying Regulatory Expectations

One point of clarification that is needed from health authorities and regulatory agencies requesting post-treatment data from oncology trials is the objective of the collection and analysis of these data. The EMA’s reflection paper on collecting PROs in oncology appears to be most aligned with a treatment efficacy objective (i.e., in a controlled setting) with the focus on theoretical equipoise as the justification for collecting these data. However, due to problems associated with collecting post-treatment data in oncology studies identified in the first section, post-treatment data would appear to lend themselves best to treatment effectiveness analyses (i.e., in a real-world setting). Per-protocol analyses are one solution to LTFU protocol deviations; however, these analyses may result in the comparison of groups that are not comparable (due to the exclusion of ITT patients). In addition, the loss of data due to attrition and lack of treatment attribution due to treatment crossover likely justify the exclusion of these data as part of any ITT efficacy analysis. While advanced modeling techniques, such as instrumental variable models and the use of propensity scores, can model treatment crossover and censored data, they are not commonly used for efficacy analysis (which are focused on actual observed data). Moreover, these types of modeling procedures make strict assumptions about the data that, in some cases, are untestable (e.g., treatment group assignment is not associated with protocol compliance). Given these complexities, post-treatment PRO data are likely best treated as preliminary effectiveness data. An alternative approach would be to simply analyze the post-treatment data descriptively without the demands of inferential statistics. While no conclusions of treatment superiority could be drawn in such cases, general trends could be observed and hypotheses for future real-world effectiveness studies could be generated. Hence, agencies requiring or recommending that sponsors collect post-treatment or post-progression data need to provide clarity for how these data are intended to be analyzed and interpreted, while acknowledging the limitations of using such data for treatment comparisons.

## Conclusion

As the outcomes of treatment drive the overall health and QOL of patients with cancer, it is important to collect data both during and after the treatment period in an oncology clinical trial. These data may be collected as part of cohort extension or registry studies to describe long-term outcomes of study participants after concluding their cancer treatment. While post-treatment PRO data may be expected to satisfy regulatory and certain payer expectations, significant practical barriers exist for the efficient incorporation of these data into oncology clinical trials, such as subject attrition, protocol deviations, and treatment crossover. This commentary discusses these challenges and offers potential solutions to help researchers when designing trials to include post-treatment PRO assessments (Table 1).

**Table 1:** Challenges and Solutions to Long-term Follow-up PRO Assessment

Challenge	Solution
Subject attrition	• Collect LTFU data from a subset of participants • Employ modes of PRO administration that can be used at the participant’s home • Conduct assessments more frequently during the start of the follow-up period • Consider reporting LTFU PRO data descriptively rather than using longitudinal statistical modeling
Protocol deviations	• Treat LTFU data as evidence for effectiveness rather than for efficacy • Consider reporting LTFU PRO data descriptively rather than using longitudinal statistical modeling
Treatment crossover/treatment switching	• Treat LTFU data as evidence for effectiveness rather than for efficacy • Consider reporting LTFU PRO data descriptively rather than using longitudinal statistical modeling
Clinical utility of LTFU PRO data	• Administer post-treatment assessments for the same frequency and duration as the on-treatment assessments • Determine how LTFU data will be analyzed and reported prior to protocol design and choose an assessment venue that will generate data that can be analyzed in that way
Cost	• Collect LTFU data from a subset of participants • Consider different options for assessment venue (e.g., passive follow-up via registries vs direct, active follow-up via cohort extension) • BYOD

*BYOD* bring your own device, *LTFU* long-term follow-up, *PRO* patient-reported outcome

The plan for collecting LTFU data should involve multiple functions in the design of the protocol. In particular, the clinical trial statisticians will need to take care when defining the LTFU estimands, as LTFU data offer numerous opportunities for the observation of intercurrent events. For example, the start of new anticancer treatment is an intercurrent event that can be omitted from the statistical model (e.g., a treatment policy strategy), used for censoring (e.g., a while-on-treatment strategy), or addressed in statistical modeling, such as multiple imputation (e.g., a hypothetical strategy). It is essential to clearly define the LTFU study objectives in the protocol, including well-defined estimands for the statistical analysis, as well as to document limitations associated with the LTFU data-collection approach. Furthermore, communications with health authorities and regulatory agencies on the LTFU study design and analysis should occur as early as possible to ensure that the PRO data to be collected offer an opportunity to properly evaluate the intended scientific research question of interest.

Overall, the incorporation of post-treatment PRO assessments is a resource-intensive task requiring clear objectives for how the data will be analyzed and interpreted by both sponsors and regulators. Incorporating PRO data collection via electronic modalities (e.g., smartphone, web) may be a less expensive and more feasible option for incorporating long-term follow-up, reducing the frequency of manual study staff follow-up and expensive clinic visits. Analytical techniques will likely rely on descriptive and model-based statistics of PRO outcomes, and conclusions about treatment differences will likely be limited to preliminary findings of effectiveness (instead of efficacy).
